# Railway Carriages and Infection

**Published:** 1900-09-22

**Authors:** 


### RAILWAY CARRIAGES AND INFECTION.

Among the many so-called causes which conspire to produce a disease it is often by no means easy to assign to each its due importance. In the case of an infectious disease it is but natural that the place of honour should be given to the presence of the germ. Truly, its presence is a *causa causans*, but while willing to pay to the microbe all the honour which is its due, no greater mistake could be made than to forget that without other contributory causes the germ is impotent. Dr. Noble Jones,[1] of Chicago, has lately been making observations in regard to the presence of tubercle bacilli in the nasal secretion of a number of apparently healthy persons, and he concludes from the results of thirty-one inoculations that virulent tubercle bacilli are found in the nasal cavities of healthy persons in the ordinary walks of life. Strauss long ago showed that the presence of virulent tubercle bacilli could be constantly demonstrated in the nasal cavities of those who, being non-tuberculous themselves, were in attendance on tuberculous persons. Turning to another disease, in the July number of the *Revue des Maladies de l'Enfance* there is an article describing two series of investigations by Kober in relation to the presence of virulent diphtheria bacilli in the throats of healthy persons. In the first of these he shows, as has long been known, that in a considerable proportion of those who, although healthy, were known to have been recently in contact with patients suffering from diphtheria, the bacilli were found in the throat. In the second series he further showed that, even among those who were not known to have recently come in contact with any diphtheria case, a diphtheria-like bacillus could now and again be isolated. There is nothing very new in this, but its interest is revived by a question brought up at the recent Congress of the Royal Institute of Public Health in Aberdeen in regard to the dangers to health arising from the dirty condition of many railway carriages. There is every reason to believe that these are a danger, and that people must now and again become infected with various diseases from being shut up in their stuffy atmosphere. But we must not jump to the conclusion that they are a very great danger, for here we have the fact before us that people may carry about sources of infection actually within themselves and yet not become infected. Susceptibility is of quite as much importance as the microbe, and, indeed, when one is told that, within this island alone, 1,100 millions of passenger journeys are made in railways every year, and that each passenger, in a second or third class carriage "is brought into actual or potential contact with 50,000 travelling companions," although we may admit that "it is impossible to deny that this implies a very considerable risk of disease," we nevertheless cannot but feel that the very immensity of the figures minimises their importance. In fact it is quite open to people to take the facts stated as pointing in exactly the opposite direction to the one intended, and to maintain that, if such an enormous disproportion exists between the cases in which the seed is sown and those in which it takes root, the germ is far from being the predominent factor in tne causation of disease.

[1] New York Med. Rec., Aug. 25.

